# The Influence of Transtibial Prosthesis Type on Lower-Body Gait Adaptation: A Case Study

**DOI:** 10.3390/ijerph20010439

**Published:** 2022-12-27

**Authors:** Yosra Cherni, Simon Laurendeau, Maxime Robert, Katia Turcot

**Affiliations:** 1 Department of Rehabilitation, Laval University, Quebec City, QC G1V 0A6, Canada; 2 Centre for Interdisciplinary Research in Rehabilitation and Social Integration (Cirris), Quebec City, QC G1M 2S8, Canada; 3 Department of Kinesiology, Laval University, Quebec City, QC G1V 0A6, Canada

**Keywords:** prosthetic devices, artificial limbs, gait rehabilitation, gait symmetry, transtibial amputation

## Abstract

Gait parameters are altered and asymmetrical in individuals with transtibial amputation. The purpose of this study was to evaluate and compare the effect of four different prosthetic feet on lower-limb biomechanics during gait. A 34-year-old man with transtibial amputation performed four gait analysis sessions with four foot–ankle prostheses (Variflex, Meridium, Echelon, and Kinterra). Kinematic and kinetic parameters and gait symmetry were analyzed in different prosthetic conditions. The type of prosthesis had little effect on the participant’s spatiotemporal parameters. Throughout the stance phase, increased hip angle, reduced knee flexion and ankle dorsiflexion were observed in the amputated leg. For kinetic parameters, reduced propulsive force (SI = 0.42–0.65), reduced knee extension moment (mainly during Echelon and Kinterra conditions, SI = 0.17 and 0.32, respectively), and increased knee abduction moment (mainly during the Variflex and Meridium, SI = 5.74 and 8.93, respectively) were measured in the amputated leg. Lower support moments were observed in the amputated leg as compared to the unaffected leg, regardless of the type of prosthesis (SI = 0.61–0.80). The prostheses tested induced different lower-limb mechanical adaptations. In order to achieve the clinical goal of better gait symmetry between lower limbs, an objective gait analysis could help clinicians to prescribe prosthetic feet based on quantitative measurement indicators to optimize gait rehabilitation.

## 1. Introduction

Lower limb amputations can result from different etiology (e.g., traumatic, vascular, congenital, infection, etc.). One of the most common amputations in lower limbs is transtibial amputation (TTA) [1,2]. TTA occurs below the knee joint and deprives patients of the foot–ankle complex. The loss of the ankle and foot significantly alters functional capacities and requires adaptation strategies to perform daily life activities, including walking and standing [3].

Compared to healthy individuals, individuals with TTA exhibit a large variety of functional limitations during gait due to the mechanical constraints imposed by their prosthesis [4,5]. These limitations are manifested mainly by reduced walking speed [6], less power generated in the amputated leg during the push-off phase [7], and increased inter-limb asymmetry in terms of spatiotemporal, kinematic and kinetic parameters (e.g., range of motion (ROM), external joint moments, ground reaction forces) [5]. Therefore, individuals with TTA must adapt their body-mechanical strategies to compensate for these functional limitations and to ensure forward body progression during the swing phase. In addition, a risk of falling has also been recognized and associated with these functional limitations in individuals with TTA [8]. Because of these acquired gait body-mechanical strategies and asymmetries, individuals with TTA are more likely to develop secondary physical conditions (e.g., knee osteoarthritis, back pain, balance deficits), which can affect social participation and quality of life [9,10]. Five research action plans have been identified by stakeholders, including the importance of improving mobility which can result in decreasing functional limitations and promoting physical activity in this population [11].

In the last decades, numerous ankle–foot prosthetic categories have been developed to address functional limitations in individuals with TTA. Despite their different mechanisms of action, the goal of these prostheses is to provide some qualities of the natural ankle–foot function to improve gait performances and reduce the risks of tripping and falling [12]. While the majority of passive prosthetic ankle–foot components include energy-storing and return prosthetic feet (e.g., Variflex), they do not incorporate an articulating ankle joint [13]. Over time, the ankle–foot component design has improved, with passive hydraulic ankle–foot prosthesis to provide greater ROM and improved toe-clearance (e.g., Echelon and Kinterra) [12] and microprocessor-controlled prosthesis to automatically adapt to different types of walking and to provide powered push-off (e.g., Meridium) [14]. However, the effect of these different types of prosthetic foot–ankle components on full lower-body gait adaptation has never been compared. The aim of this case study was to evaluate the effect of four types of prostheses on body-mechanical strategies on the lower limbs in a participant with TTA by comparing the biomechanical variables influenced by each prosthesis type. Since the microprocessor-controlled prosthetic feet have been designed to increase ROM of the ankle and to provide powered push-off in the amputated leg [14], it is expected that the Meridium would lead to better legs symmetry when compared to other types of prosthesis. As the gait rehabilitation process is a patient-specific treatment, a case study might be appropriate to better understand the effect of different prostheses.

## 2. Materials and Methods

### 2.1. Case Description

A healthy and active man with unilateral TTA (age = 34 years; weight = 81.8 kg; height = 1.7 m) participated in this study within the framework of his medical supervision at the Quebec Institute of Rehabilitation in Physical Deficiency. The surgical procedure for the participant’s below-the-knee amputation occurred at the age of 2 months old due to a peripheral vascular disease. The stump length is approximately 8 cm, which was measured from the tibial tubercle. The participant has a full-time office job and is able to walk with a prosthesis without an additional walking aid. The participant provided written informed consent before participating in this Institutional Review Board-approved study (2016-489).

### 2.2. Experimental Protocol and Data Collection

**Figure 1:**
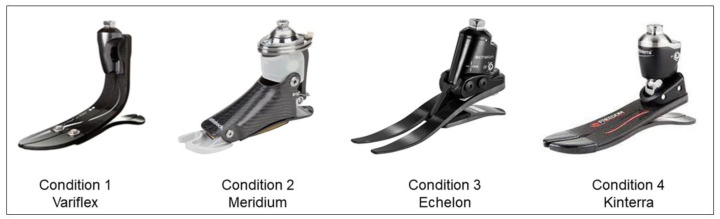
The four tested prostheses. Variflex LP (Össur, Reykjavik, Iceland): ESR prosthesis with a carbon foot. Meridium (Ottobock, Duderstadt, Germany): a microprocessor-controlled prosthetic that automatically adjusts to different types of walking, changes in inclination and different walking speeds. Echelon VT (Chas. A Blatchford & Sons, Basingstoke, UK): A dynamic carbon fiber foot comprising independent toe and heel springs with a hydraulic self-aligning ankle. Kinterra (Freedom Innovations, Morgan, CA, USA): A hydraulic articulation combined with an energy-storage-and-return foot. The specificity of each prosthesis is detailed in Table S1.

Based on the participant’s needs, the choice of prostheses was made following a discussion between two experienced prosthetists, one of which who has been working with the participant for over 10 years. The individual’s functional capacities, activities of daily living and type of employment are best aligned with four specific foot–ankle prostheses (i.e., Variflex, Meridium, Echelon and Kinterra). The descriptions of different prostheses are reported in Figure 1 and Table S1. To compare the effect of prostheses type on gait parameters, the participant performed four gait analysis sessions at a self-selected speed with the four prostheses. All prostheses were checked for alignment by an experienced prosthetist. For each prosthesis, a familiarization period of a minimum of 10 days was completed (i.e., more than a year with Variflex; 12 days with Meridium; 10 days with Echelon; 11 days with Kineterra). The same socket was used with each prosthesis.

Gait analysis: A 10-camera motion capture system (Vicon, Vantage 5, Oxford, UK) was used to collect the trajectories of reflective markers for kinematic data (sampled at 100 Hz) and four strain gauge force platforms embedded in the laboratory walkway (AMTI^®^, OR6, Watertown, MA, USA) were used for kinetic data (sampled at 1000 Hz). For the experimental calibration procedures, 72 reflective markers were placed bilaterally (and symmetrically on the prosthesis) using a six degrees of freedom model based on the International Society of Biomechanics recommendations for the lower limbs and the trunk [15]. The participant was asked to walk on a 10-m walkway at his self-selected comfortable speed wearing his own neutral shoes (same for each visit). To avoid fatigue, the participant could rest if needed. Finally, successful trials were those comprising full contact on a single force platform and when all markers were viewable.

Prosthetic Profile of Amputee questionnaire: At the end of each evaluation session, the Prosthetic Profile of Amputee (PPA) questionnaire was completed by the participant in order to identify the factors related to each prosthesis use (i.e., comfort, adaptation, appearance, etc.) [16]. However, because the PPA is an extensive, lengthy questionnaire, only items about the prosthesis were selected for this study.

### 2.3. Data Analysis

Kinematic and kinetic parameters: Marker trajectories were tracked and analyzed using Nexus version 2.6.1 (Vicon Motion Systems, Oxford, UK). The Motion Monitor software (Innovative Sports Training, Chicago, IL, USA) was used to process spatiotemporal, kinematic, and kinetic data. Kinematics from the ankle, knee, hip, and pelvis were calculated using an XYZ Cardan–Euler sequence. The pelvis segment angles were calculated with respect to laboratory coordinate systems, while the hip, knee and ankle angles and moments were calculated relative to the proximal segment. The inverse dynamic was used to calculate net external moments of hip, knee, and ankle joints (all normalized by body mass). The vertical and anteroposterior ground reaction forces were measured by two piezoelectric force plates placed in the middle of the 10 m walkway of the laboratory. For each prosthetic condition, 4 to 8 gait cycles per leg were selected for analysis using a customized software application (Moveck Solution Inc., Quebec, QC, Canada).

Total Support Moment: As defined by Winter [17], the total support moment (TSM) was calculated as the sum of extensor moments of the ankle, knee, and hip at the entire stance phase, as well as the subphases of stance.

Gait symmetry: The symmetry index (SI) compared the kinematics and kinetics of the amputated leg to the unaffected leg for each type of prosthesis tested. The symmetry index was calculated during the stance phase of the gait cycle for each subject and was calculated for each condition as follows [18,19]:

$$\mathrm{SI}\;=\frac{V_{AL}}{V_{UL}}$$

with *V_AL_* and *V_UL_* representing the values of the gait parameters, calculated, respectively, for the amputated leg and the unaffected leg. When no differences were measured between the two limbs, SI equaling one, lower, or higher values indicated asymmetry in the gait parameters. Using a ratio equation to describe asymmetry is recommended when there is an identifiable affected side so that the results are relatively easy to interpret [20,21].

## 3. Results

### 3.1. Prosthetic Profile of the Amputee

The results of prosthesis use section of PPA are displayed in Table 1. Overall, the participant was more satisfied with the use of the Echelon in terms of comfort, weight, appearance, and gait appearance compared to other prostheses. In terms of comfort, the Meridium was less comfortable than other prostheses (Table 1).

### 3.2. Kinematic Parameters

Spatiotemporal parameters are summarized in Table 2. Overall, the tested prostheses allowed a symmetry of the spatiotemporal parameters ranging from 0.94 to 1.03, except for the step length when walking with a Kinterra prosthesis (SI = 1.25).

**Figure 2:**
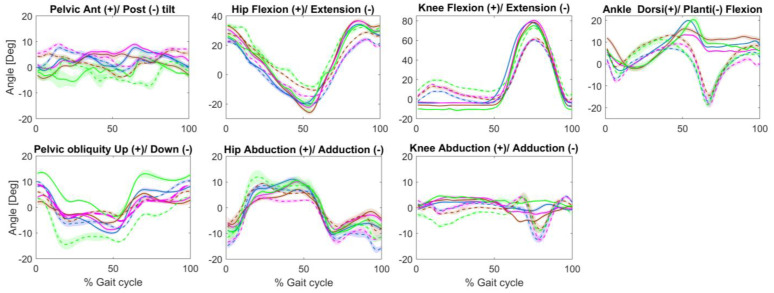
Joint angles in the sagittal and frontal plane during walking in four prostheses conditions: Variflex (blue), Meridium (brown), Echelon (magenta), and Kinterra (green). The kinematic of the amputated leg is presented in a continuous line, and that of the unaffected leg is presented in a dotted line. The shaded region denotes the standard deviation.

*Joint angles results* are displayed in Table 2 and Figure 2. In the sagittal plane, an increase in pelvic retroversion was observed during the stance phase when walking with the Kinterra prosthesis provoking a greater hip flexion in the unaffected leg compared to other prosthetic conditions. Knee extension was observed in the amputated leg throughout the stance phase in all four prosthetic conditions, but more prominently with the Kinterra prosthesis (knee extension = 11.49°). In all prosthetic conditions, increased ankle dorsiflexion for the amputated leg with an absence of push-off was observed as compared to the unaffected leg. In terms of gait symmetry, walking with the Variflex and Kinterra prostheses induced lower ROM symmetry at the pelvis (SI = 0.84 and 0.82, respectively) and at the knee (SI = 1.55 and 2.09, respectively) during the stance phase. The Meridium and Echelon prostheses tended to increase the hip ROM of the amputated leg during the stance phase (SI = 1.51 and 1.41, respectively). In the frontal plane (Figure 2), greater pelvis elevation of the amputated side was observed when walking with the Kinterra prosthesis. Furthermore, with the Kineterra prosthesis, the ankle maintained an adducted position throughout the gait cycle.

### 3.3. Kinetic Parameters

**Figure 3:**
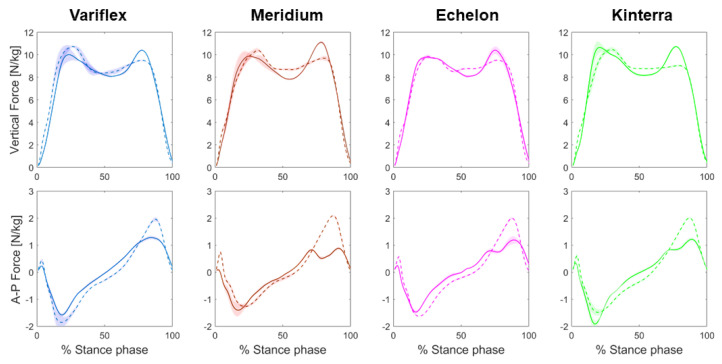
Mean vertical and anterior–posterior components of the ground reaction force patterns during stance at different prostheses’ conditions. Forces of the amputated leg are presented in a continuous line, and that of the unaffected leg is presented in a dotted line. The shaded region denotes the standard deviation.

The results of the ground reaction force from the amputated and unaffected legs during different prostheses conditions are shown in Table 3 and Figure 3. Regarding the vertical component of the ground reaction force, differences between the legs reflected mainly higher peak force in the late stance phase for the amputated leg as compared to the unaffected leg (SI = 1.09–1.19). This difference in the second vertical peak force was especially apparent in the Meridium and Kinterra conditions (SI = 1.15 and 1.19, respectively). For the anterior–posterior component of the ground reaction force, the amputated leg had lower peak propulsive forces than the unaffected leg (SI = 0.42–0.65) during all the prosthetic conditions. The difference in the peak of propulsive forces between the amputated and unaffected legs was greater in the Meridium condition (SI = 0.42).

**Figure 4:**
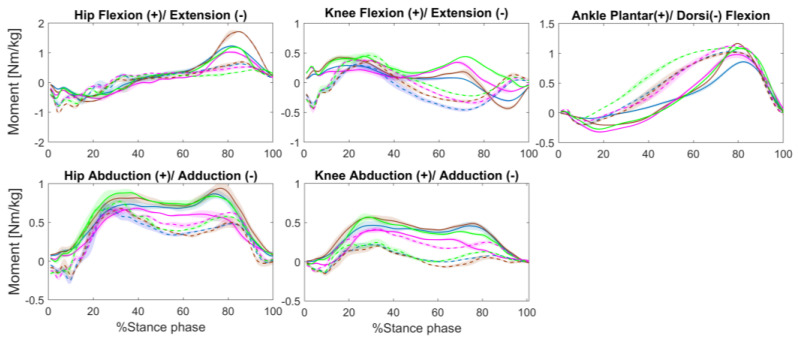
Joint moment means waveforms for four prosthetic conditions subject groups: Variflex (blue), Meridium (brown), Echelon (magenta), and Kinterra (green). The kinematic of the amputated leg is presented in a continuous line, and that of the unaffected leg is presented in a dotted line. The shaded region denotes the standard deviation.

Regarding joint moments, the hip extension moment decreased at late stance in the amputated leg in all the tested conditions (Figure 4). Relative to the unaffected leg, a lack of knee extension moment was observed in the amputated leg during stance. Compared to the unaffected leg, ankle plantarflexion moment was decreased during stance in the amputated leg when walking with the Variflex and Echelon prostheses (SI = 0.83–0.89). In regards to the frontal plane, all prostheses, with the exception of the Echelon, displayed increased knee abduction moment in the amputated leg at early (SI = 2.14–2.81) and late stance (SI = 3.17–8.93). However, the Echelon prosthesis allowed a between-limbs asymmetry at early (SI = 0.98) and late stance (SI = 1.22).

**Figure 5:**
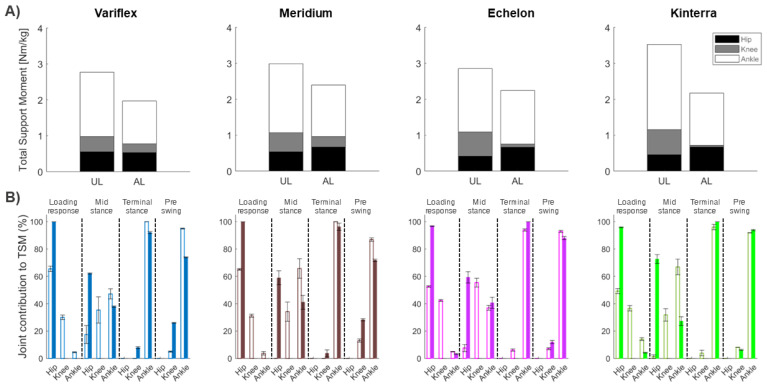
(**A**) Mean total support moment at the four conditions during the entire stance phase. (**B**) Individual joint contributions to total support moment as a function of the sub-phases of the stance phase. Results of the amputated leg (AL) are presented in filled bars, and that of the unaffected leg (UL) are presented in bars with colored borders: Variflex (blue), Meridium (brown), Echelon (magenta), and Kinterra (green).

The combined effect of the net extensor moments of the hip, knee, and ankle joints are reflected by the TSM, as shown in Table 1 and Figure 5. Overall, the participants generated lower support moments in the amputated leg compared to the unaffected leg, regardless of the type of prosthesis (SI = 0.61–0.80). The TSM of the amputated leg was lower with the Variflex prosthesis compared to other prostheses. In terms of joint contributions to TSM, more substantial differences between limbs were apparent at the knee (Figure 5), particularly in the Echelon (contribution = 4% in the amputated leg vs. 24% in the unaffected leg) and Kinterra conditions (contribution = 2% in amputated leg vs. 20% in the unaffected leg).

## 4. Discussion

The objective of this case study was to compare the effect of four prostheses on biomechanical gait parameters in one participant with a TTA. A single-subject design was used in this study to preserve the participant-specific information as previously described in other studies [22,23]. Overall, the results of this study showed a disparity in biomechanical adaptations between the different prostheses. However, there are three major findings to be addressed. First, throughout the stance phase, increased hip ROM and reduced knee flexion and ankle dorsiflexion were observed in the amputated leg when walking, which highlighted specific asymmetries induced by each prosthesis. Second, reduced propulsive force (IS = 0.42–0.65), reduced knee extension moment (mainly in the Echelon and Kinterra conditions, IS = 0.17 and 0.32, respectively), and increased knee abduction moment (mainly in the Variflex and Meridium conditions, IS = 5.71 and 8.93, respectively) were denoted in the amputated leg. Third, the participant generated lower support moments in the amputated leg when compared to the unaffected leg, regardless of the prosthetic type.

### 4.1. Effect of Different Prostheses on Lower Limb Kinematics

The Echelon and Meridium prostheses induced greater hip ROM on the amputated side compared to the unaffected leg (SI = 1.41–1.54), causing higher asymmetry. This result was likely compensation for the reduced ROM at the ankle when walking with these prostheses. On the other hand, important knee ROM asymmetries were observed mainly in the Variflex and Kinterra conditions (SI = 1.55–2.09, respectively). Indeed, the residual knee remained in extension throughout the stance phase in the four prosthetic conditions but was more prominent in the Kinterra condition (knee extension = 11.49°). This could be due to insufficient socket flexion (foot plantar flexed) [24]. In addition, the loss of knee flexion in the amputated leg may require body-mechanical compensations, which could affect load bearing in the intact knee. For example, the observed reduction in residual knee flexion angle during midstance could be evidence of a quadriceps-avoidance gait. Previous studies [25,26] reported compensatory muscle activity in individuals with TTA during walking, namely asymmetry in intact versus residual knee flexor/extensor activity. In general, abnormal knee kinematics are linked to the onset of knee osteoarthritis [27]. Similarly, a notable difference between legs was observed at the ankle joint, particularly in the late stance and early swing when the plantar flexion movement within the prosthesis was substantially lower than that of the intact ankle joint, regardless of the prosthetic condition. Increased ankle dorsiflexion of the amputated leg is a strategy that facilitates minimal toe clearance, which is required to prevent tripping and falls. However, the lack of ankle plantarflexion, as observed in all the conditions, affected the propulsion. These kinematic deviations were coherent with the observed differences between legs in terms of joint moments and ground reaction forces.

### 4.2. Effect of Different Prostheses on Lower Limb Kinetics

In terms of joint moments, large asymmetries were present in maximum hip and knee extensor moments during gait, suggesting that muscle coordination and braking/propulsion effort may be altered for the residual leg with different prostheses. Moreover, the knee adduction moment is an important parameter, as the peak knee adduction moment is related to the initiation and progression of knee OA [28]. In the present study, the participant showed higher knee abduction moment in the residual knee, which led the intact knee to a moment tending towards adduction when walking with the Meridium and Kinterra prostheses as compared to other conditions. However, the Echelon tended to induce similar abduction moments at both intact and residual knees. For the vertical ground reaction forces, all conditions reflected the standard pattern with the double peaks. Our results showed that the magnitude of the first peak was quite similar on both sides for all prostheses used. This was not the case for the second peak that increased in the amputated leg compared to the unaffected leg (mainly when walking with the Meridium or Kinterra). One possible explanation is that the participant preferred to load his weight on the unaffected leg as a protective gait strategy. For the AP ground reaction forces, the propulsive peak was lower by 34% to 58% in the amputated leg (mainly when walking with the Meridium prosthesis). This result agrees with kinematics results. This showed a more vertical leg orientation (i.e., by an increased hip and knee extension in the amputated leg) which would result in reduced AP ground reaction forces as well as an inability of the participant to modulate the ankle plantar flexor moment during stance (Figure 4). These deviations together contribute to a less propulsive gait [29].

The combined effect of the net extensor moments of the hip, knee, and ankle joints was reflected by the TSM. In general, a smaller TSM was observed for the amputated leg as compared to the unaffected leg. This result may underline the participant’s inability to compensate for the loss of ankle extensor muscles (i.e., Soleus and Gastrocnemius), which then contributes to vertical support throughout a single stance [30]. During the mid-stance phase, Soleus and Gastrocnemius together ensure support and forward progression of both the leg and trunk [30,31]. Thus, the loss or impairment of Soleus and Gastrocnemius force generation would negatively impact gait stability. In addition, the results of this study showed a small contribution of the knee extensor moment to TSM in comparison with the ankle and hip joints in both legs. These results are consistent with previous studies that showed a dominant contribution of the ankle moment in support and propulsion during locomotion [17,32]. However, this effect was amplified when walking with Echelon and Kinterra prostheses, for which the knee moment of the amputated leg never became extensors for all of the stance phases. Thus, a net knee extensor moment was not crucial to the development of the necessary extensor support moment on the amputated leg when walking with these prostheses. Similarly, Sanderson et al. [33] showed that individuals with TTA amputation were able to generate an extensor support moment on the amputated leg in the absence of the knee extensor moment. As shown by our results, the decrease in knee contribution seemed to be mainly compensated by the hip extensor moment. In summary, the results of this case study suggest that depending on the type of prosthesis, the participant adopts different support strategies in the unaffected and amputated legs.

### 4.3. Clinical Implications

In summary, locomotor adaptations were different from one prosthesis to another. However, less proximal compensations at the hip and knee may be key factors in terms of participant preference. The present results demonstrated a better symmetry of lower limb joint moments (except for knee extensor moment) and ground reaction forces when using the Echelon prosthesis. However, specific to this case, the use of the Kinterra prosthesis resulted in higher pelvis and knee ROM asymmetries, which may produce important proximal compensations in the unaffected leg. This result may be explained by the fact that this prosthesis offered less control of the prosthetic ankle and, therefore, required more proximal compensations. Similarly, large asymmetries were observed in lower limb extension moments during gait, especially when walking with Echelon and Kinterra prostheses, suggesting that muscle coordination and braking/propulsion effort may be more affected in the amputated leg with these prostheses. Compared to the unaffected leg, the Variflex and Meridium prostheses produced a significant increase in residual knee abduction moments that could pose an important risk factor for knee osteoarthritis. Finally, personal preferences, needs and comfort, as well as the prosthesis cost-benefit, are generally also important factors to consider. For example, the Meridium, which had the highest cost among the tested prostheses, seemed to be the least comfortable prosthesis for the participant (Table 1). However, given the participant profile (low displacement with the prosthesis) and specificity of this prosthesis (a microprocessor-controlled prosthesis, Table S1), a longer familiarization period would be necessary to overcome this discomfort. Finally, within the framework of this study, the Echelon was the better answer according to the needs of the participant in terms of comfort, gait adaptation, and cost.

### 4.4. Study Limitations

There are some limitations in this study that need to be acknowledged. First, only one case was reported, which reduces the external validity of the results. However, this design was used to generate hypotheses for further studies to verify if these results would translate to a larger population. Second, the participant is a healthy and active individual, which may limit the choices of prostheses. A study with older and/or more sedentary participants would better compare the contribution of the microprocessor-controlled prosthetic foot versus a foot with energy storage and return. Third, the current results were also limited to walking tasks. Further studies assessing the effect of different types of prosthesis during various functional tasks (e.g., sit to stand, stair ascent and descent, physical activity) are needed to better understand their functional contribution in a real-life situation. Fourth, muscle activity during gait was not recorded, which limits the understanding of the compensatory strategies induced by the different prostheses.

## 5. Conclusions

Gait impairments in individuals with TTA can be attributed to either mechanical constraints imposed by the prosthesis or patient-specific factors. The results of this case study showed a disparity in biomechanical adaptations between the different prostheses in terms of joint angles, moments, and contributions. Finally, objective and multifactorial determinants (e.g., environmental, contextual, workspace, etc.) may be needed for prescribing prosthetic feet. Further studies should evaluate the impact of these prostheses on gait and various activities of daily living in a larger number of participants with different functional levels.

## Figures and Tables

**Table 1:** The prosthesis section results of the PPA questionnaire.

Prosthesis Section	Subsection	Variflex	Meridium	Echelon	Kinterra
**Question 1**	Comfort	4	5	5	5
	Appearance	5	5	5	5
	Weight	5	3	5	5
	Appearance of your gait	4	5	5	4
**Question 2**	Amputation	5	5	5	5
	Prosthesis	5	3	5	4
**Question 3**		None	Insecurity walking in a small environment due to the inability of the leg to react quickly.	None	Insecurity when making turns or picking up objects if I must rotate.
**Question 4**		Changed for a different type of prosthesis	Changed for a different type of prosthesis	Changed for a different type of prosthesis	Changed for a different type of prosthesis
**Question 5**	Able to quickly give you an appointment?	Yes	Yes	Yes	Yes
	Located sufficiently close to your home?	Yes	Yes	Yes	Yes

**NOTE: Question 1**: Four characteristics concerning your prosthesis are listed below. Please indicate your degree of satisfaction with each one of these characteristics. **Question 2**: The adaptation (in the sense of «GETTING used to…»): getting used to the amputation and to the prosthesis may be more difficult for some people than for others, and this adaptation is not always easy to evaluate. After examining the given choices of possible answers, please indicate the answer that best describes your actual adaptation to your… **Question 3:** At the present time, when you wear your prosthesis, does it cause you… **Question 4**: Since the time you completed your rehabilitation program, has your prosthesis been… **Question 5:** In your opinion, is your prosthesis laboratory.

**Table 2:** Kinematic and kinetic outcomes during the four conditions. Values are expressed as mean (SD).

	Amputated Leg				Unaffected Leg			
	Variflex	Meridium	Echelon	Kinterra	Variflex	Meridium	Echelon	Kinterra
**Spatiotemporal parameters**								
Speed (m/s)	1.20 (0.03)	1.15 (0.03)	1.13 (0.02)	1.15 (0.03)	1.19 (0.02)	1.16 (0.02)	1.13 (0.02)	1.17 (0.03)
Step length (m)	0.67 (0.03)	0.69 (0.02)	0.67 (0.01)	0.69 (0.02)	0.67 (0.03)	0.70 (0.01)	0.67 (0.01)	0.55 (0.28)
Cadence (step/min)	105.30 (2.36)	100.02 (1.75)	101.13 (0.90)	101.05 (0.42)	105.16 (1.34)	100.85 (1.20)	101.41 (1.03)	101.94 (2.15)
Stance (%)	62 (0.82)	61 (1.03)	63 (0.30)	65 (4.02)	64 (0.71)	65 (0.93)	66 (0.37)	63 (0.75)
**Joint angles**								
Pelvis Ante/Retroversion (°)								
- ROM at stance	7.67 (1.11)	9.56 (1.47)	8.92 (0.54)	8.51 (3.02)	9.17 (1.10)	8.98(1.29)	8.74 (1.01)	10.28 (1.37)
- Peak anteversion at stance	6.40 (1.20)	5.23 (0.67)	8.39 (0.60)	2.18 (1.92)	7.62 (0.85)	5.21 (0.85)	9.08 (0.53)	3.47 (1.43)
- Peak retroversion at stance	−1.26 (0.46)	−4.32 (0.95)	−0.53 (0.72)	−6.33 (1.30)	−1.55 (0.72)	−3.78 (0.57)	0.34 (0.39)	−6.81 (0.99)
Hip Flexion/Extension (°)								
- ROM at stance	44.66 (2.13)	59.08 (2.20)	53.04 (1.15)	47.74 (7.57)	42.37 (1.01)	38.48 (1.09)	37.65 (1.32)	41.83 (1.11)
- Peak flexion at stance	25.18 (1.09)	33.25 (1.95)	30.77 (1.27)	26.62 (4.65)	22.35 (1.08)	27.88 (1.21)	22.80 (1.18)	33.59 (1.05)
- Peak extension at stance	−19.47 (1.32)	−25.83 (1.42)	−22.26 (1.19)	−21.12 (4.70)	−20.02 (0.40)	−10.60 (1.01)	−14.85 (0.67)	−8.24 (1.12)
Knee Flexion/Extension (°)								
- ROM at stance	55.37 (2.23)	37.46 (2.53)	44.17 (0.94)	59.24 (4.45)	35.74 (3.5)	33.78 (2.48)	33.92 (3.50)	28.33 (3.31)
- Peak flexion at stance	51.24 (2.30)	30.48 (2.50)	40.15 (0.93)	47.74 (3.72)	31.27 (3.98)	33.34 (2.36)	35.58 (2.15)	36.23 (2.94)
- Peak extension at stance	−4.13 (0.27)	−6.98 (0.23)	−4.02 (0.17)	−11.49 (1.32)	−4.46 (1.70)	−0.45 (1.24)	1.66 (1.64)	7.90 (1.17)
Ankle Flexion/Extension (°)								
- ROM at stance	22.92 (0.53)	18.23 (0.81)	14.82 (0.37)	22.02 (0.92)	21.87 (2.26)	25.23 (1.35)	24.63 (1.14)	25.06 (1.26)
- Peak dorsi-flexion at stance	19.92 (0.36)	16.08 (1.04)	13.35 (0.36)	20.37 (1.29)	7.05 (0.54)	13.19 (0.68)	9.27 (0.75)	11.08 (1.63)
- Peak plantar flexion at stance	−3.00 (0.22)	−2.15 (1.25)	−1.47 (0.26)	−1.65 (1.61)	−14.83 (2.33)	−12.04 (1.33)	−15.36 (1.28)	−13.98 (0.38)
**Kinetic parameters**								
Ground reaction force (N/kg)								
- 1st vertical peak	10.01 (0.61)	9.88 (0.29)	9.79 (0.20)	10.65 (0.46)	10.73 (0.09)	10.40 (0.22)	9.84 (0.18)	10.47 (0.21)
- 2nd vertical peak	10.40 (0.07)	11.11 (0.13)	10.41 (0.35)	10.73 (0.12)	9.50 (0.07)	9.70 (0.21)	9.52 (0.06)	9.04 (0.11)
- AP Braking peak	−1.58 (0.06)	−1.41 (0.23)	−1.47 (0.08)	−1.92 (0.12)	−1.86 (0.23)	−1.27 (0.05)	−1.63 (0.01)	−1.49 (0.17)
- AP Propulsion peak	1.28 (0.07)	0.88 (0.06)	1.19 (0.15)	1.22 (0.05)	1.96 (0.08)	2.10 (0.02)	2.00 (0.05)	2.01 (0.03)
Joint moments (Nm/kg)								
- Peak hip extension	−0.46 (0.01)	−0.63 (0.06)	−0.53 (0.08)	−0.53 (0.03)	−0.75 (0.10)	−1.02 (0.02)	−0.81 (0.07)	−0.79 (0.02)
- Peak knee extension	−0.30 (0.02)	−0.43 (0.03)	−0.15 (0.02)	−0.07 (0.01)	−0.46 (0.03)	−0.43 (0.01)	−0.46 (0.06)	−0.42 (0.03)
- Peak ankle plantarflexion	0.86 (0.02)	1.16 (0.01)	0.98 (0.06)	1.08 (0.02)	1.03 (0.01)	1.02 (0.01)	1.13 (0.01)	1.10 (0.01)
- Peak hip abduction at early stance	0.74 (0.08)	0.82 (0.07)	0.68 (0.03)	0.88(0.04)	0.67 (0.07)	0.68 (0.01)	0.66 ((0.05)	0.77 (0.02)
- Peak hip abduction at late stance	0.87 (0.06)	0.94 (0.09)	0.62 (0.05)	0.84 (0.02)	0.48 (0.01)	0.50 (0.07)	0.63 (0.01)	0.58 (0.02)
- Peak knee abduction at early stance	0.46 (0.06)	0.56 (0.03)	0.40 (0.03)	0.57 (0.04)	0.22 (0.02)	0.20 (0.01)	0.41 (0.03)	0.25 (0.05)
- Peak knee abduction at late stance	0.46 (0.05)	0.49 (0.03)	0.32 (0.01)	0.40 (0.02)	0.08 (0.01)	0.05 (0.03)	0.26 (0.02)	0.13 (0.01)
Total support moment (Nm/kg)								
- TMS	1.97 (0.01)	2.40 (0.23)	2.24 (0.02)	2.17 (0.10)	2.77 (0.23)	2.99 (0.17)	2.86 (0.11)	3.53 (0.14)
- Hip contribution	0.53 (0.01)	0.67 (0.15)	0.66 (0.04)	0.67 (0.10)	0.55 (0.06)	0.54 (0.02)	0.41 (0.03)	0.45 (0.02)
- Knee contribution	0.25 (0.02)	0.29 (0.05)	0.09 (0.01)	0.05 (0.01)	0.43 (0.05)	0.53 (0.05)	0.68 (0.03)	0.70 (0.12)
- Ankle contribution	1.20 (0.01)	1.44 (0.05)	1.49 (0.04)	1.46 (0.05)	1.80 (0.18)	1.92 (0.21)	1.77 (0.07)	2.38 (0.16)

**Table 3:** Symmetry index for kinematic and kinetic outcomes during the four conditions.

	SI-Variflex	SI-Meridium	SI-Echelon	SI-Kinterra
**Spatiotemporal parameters**				
Speed	1.01	0.99	1.00	0.98
Step length	1.00	0.99	1.00	1.25
Cadence	1.00	0.99	1.00	0.99
Stance phase	0.96	0.94	0.95	1.03
**Sagittal joint angles**				
- Pelvis ROM at stance	0.84	1.06	1.02	0.82
- Hip ROM at stance	1.05	1.54	1.41	1.14
- Knee ROM at stance	1.55	1.11	1.30	2.09
- Ankle ROM at stance	1.05	0.72	0.60	0.88
**Kinetic parameters**				
Ground reaction force				
- 1st vertical peak	0.93	0.95	0.99	1.02
- 2nd vertical peak	1.09	1.15	1.09	1.19
- AP Braking peak	0.85	1.11	0.90	1.29
- AP Propulsion peak	0.65	0.42	0.60	0.61
Joint moments (Nm/kg)				
- Peak hip extension	0.61	0.62	0.65	0.66
- Peak knee extension	0.66	0.99	0.32	0.17
- Peak ankle plantarflexion	0.83	1.14	0.89	0.98
- Peak hip abduction at early stance	1.09	1.21	1.03	1.15
- Peak hip abduction at late stance	1.80	1.88	0.99	1.45
- Peak knee abduction at early stance	2.14	2.81	0.98	2.32
- Peak knee abduction at late stance	5.71	8.93	1.22	3.17
Total support moment				
- TMS in stance	0.71	0.80	0.79	0.61
- Hip contribution to TMS	0.96	1.25	1.61	1.47
- Knee contribution to TMS	0.57	0.55	0.13	0.07
- Ankle contribution to TMS	0.67	0.75	0.84	0.61

**NOTE:** ROM: Range of Motion; AP: Antero-posterior; TSM: Total Moment Support; SI: Symmetry Index, with a value of 1 indicating perfect, >1 indicating asymmetry with higher parameter value for the amputated leg, and values < 1 indicating asymmetry with higher parameter value for the unaffected leg.

## Data Availability

Not Applicable.

## Supplementary Materials

The following supporting information can be downloaded at: https://www.mdpi.com/article/10.3390/ijerph20010439/s1, Table S1: Description of tested prostheses.
