# Supplementary material for: The Influence of Transtibial Prosthesis Type on Lower-Body Gait Adaptation: A Case Study
Source: Int J Environ Res Public Health. 2022 Dec 27;20(1):439. doi: 10.3390/ijerph20010439 (PMC9819038; doi:10.3390/ijerph20010439)
Supplement: Supplementary file 1 [file ijerph-20-00439-s001.zip › ijerph-2046835-supplementary.pdf]

Supplementary materials

Table S1. Description of tested protheses.

	**Variflex LP (Össur)**	**Meridium (Ottobock)**	**Echelon VT (Blatchford)**	**Kinterra (Freedom Innovations)**
**Category**	Energy Storage and return foot	A microprocessor-controlled prosthetic	A dynamic carbon fibre foot comprising independent toe and heel springs with a hydraulic self-aligning ankle.	Combine a hydraulic articulation unit with an energy-storage-and-return foot
**Degrees of freedom**	The Variflex LP does not offer assisted ankle plantar/dorsal flexion. The patient must exert a force on the carbon plate which will induce an energy return.	A 4-axis mechatronic foot with hydraulic movement of the ankle. It offers 36.5 degrees of plantar-flexion and dorsal-flexion.	A hydraulic foot that offers 9 degrees of plantar-flexion and dorsi-flexion.	A hydraulic foot that offers 12 degrees of ankle mobility.
**Prosthesis specification**	This prosthesis was combined to DeltaTwist shock absorber, which also features a torsion function.	The Meridium automatically adjusts to different types of walking, changes in inclination and different walking speeds.	The Echelon VT offers shock absorption and a rotation adapter included in the foot.	The Kinterra foot was combined with a durashock. This piece of elastomer serves as a shock absorber and rotation adapter.
